# Construction of a Training Content System for New Nurses in Cancer Hospital Based on Competency

**DOI:** 10.3389/fsurg.2021.833879

**Published:** 2022-02-22

**Authors:** Miao Liu, Jingzhi Geng, Jian Gao, Zhihong Mei, Xueyan Wang, Sicong Wang, Yan Liu

**Affiliations:** ^1^Department of Gynecologic Oncology, National Cancer Center/National Clinical Research Center for Cancer/Cancer Hospital, Chinese Academy of Medical Sciences and Peking Union Medical College, Beijing, China; ^2^Department of Radiation Therapy, National Cancer Center/National Clinical Research Center for Cancer/Cancer Hospital, Chinese Academy of Medical Sciences and Peking Union Medical College, Beijing, China; ^3^Department of Internal Medicine, National Cancer Center/National Clinical Research Center for Cancer/Cancer Hospital, Chinese Academy of Medical Sciences and Peking Union Medical College, Beijing, China; ^4^Department of Thoracic Surgery, National Cancer Center/National Clinical Research Center for Cancer/Cancer Hospital, Chinese Academy of Medical Sciences and Peking Union Medical College, Beijing, China

**Keywords:** competency, cancer care, new nurse, Delphi technique, index system

## Abstract

**Objective:**

To construct a training content system for new nurses in cancer hospitals based on postcompetency and to provide guidance for clinical new nurse training.

**Methods:**

Based on literature review, semistructured interviews, and questionnaire surveys, a new draft of the nurse training content system was initially established, and 17 experts were selected to make two rounds of inquiry on the system by the Delphi method, so as to construct a new nurse training content system.

**Results:**

The effective rate of recovery of the two rounds of expert correspondence was 100%, the cooperation among experts was high, and the authoritative coefficient of experts was 0.89. The content system of new nurse training constructed included 2 first-class indexes, 5 second-class indexes, and 45 third-class indexes.

**Conclusion:**

The new nurse training content system is closely combined with clinical work, pays attention to improving nurses' competence, reflects the characteristics of nursing work in cancer hospitals, has a certain scientific and practical significance, and can provide guidance for the training of new nurses in cancer hospitals.

## Introduction

In recent years, the incidence and mortality of cancer have remained high, and the demand for cancer care has become increasingly prominent. A large number of new nurses join the cancer specialist hospital every year. The process of changing from nursing students to working as new nurses is full of challenges (1, 2). In order to meet the requirements of clinical nursing work in a short period of time, it is necessary to carry out standardized training for new nurses. The current reference to the new nurse-standardized training programs (trial), some of which are not completely applicable to cancer hospitals, and the training plan should be improved and optimized according to the nursing characteristics of cancer hospitals, for example, tumor treatment methods including surgical treatment, radiotherapy, chemotherapy, gene targeted therapy, biological treatment, etc. (3). New nurses need to understand a variety of treatment methods and adverse reaction processing, the management of related drugs, the maintenance of a variety of venous lines, the first-aid for a drug anaphylactic shock, observation and care of bone marrow suppression, prevention and care of radiation inflammation, psychological care for patients with facial defects, improvement in the hospice care for patients at the end stage, implementation of the nurse occupational protection, etc. Nursing postcompetency is based on the cognition and construction method, which reflects the ability of nurses to use various nursing skills, professional knowledge, values, critical thinking, and information as a whole (4). Multiple studies have shown that a competency-based training system can urge new nurses to improve their professional knowledge and skills, change their working attitudes, etc., and ultimately improve the level of work (5, 6). This study was designed to build a training content system for new nurses in cancer hospitals based on postcompetency, so as to guide the training of new nurses, improve their quality, and make them better qualified for the job.

## Information and Methods

### Draft Framework for Initially Constructing a New Induction Nursing Training System for Oncology Specialty

Through literature review and semistructured interviews, combined with the new nurse training status and needs of the baseline survey, the new nurse standardized training program (trial), the existing training programs, and characteristics of tumor hospitals, the draft framework of the new nurse training system in tumor hospitals based on job competency was prepared. Using the method of objective sampling, a questionnaire survey was conducted among 17 experts from a certain tertiary grade A cancer hospital. The draft framework has been revised and adjusted. Through the analysis and arrangement of the preliminary survey results, a formal draft was formed.

### Design of Expert Consultation Questionnaire

The questionnaire is divided into two parts. Part I: The core part of the expert inquiry is the new nurse based on the post-competency evaluation index system expert inquiry table, using Likert grade 5 scoring method (5 points-very important, 4 points-important, 3 points-generally important, 2 points-not too important, and 1 point-not important) to determine the importance of each index items, and put forward items or suggestions for addition or deletion. Part II: Basic information sheet of experts, including general information of experts (age, length of service, educational background, etc.); judgment basis (including theoretical analysis, work experience, references, and intuitive selection; the degree of influence was divided into large, medium, and small grades); familiarity with the content of the study (divided into six levels of very familiar, familiar, more familiar, generally familiar, less familiar, and very unfamiliar).

### Implementation of Delphi Expert Consultation

#### Number and Criteria for Selection of Experts

The number of experts depends on the content of the study, and the appropriate number of general experts is 15–50. The selected experts should have certain academic authority, professional knowledge, and clinical experience in the corresponding research fields. Inclusion criteria are as follows: a) Being engaged in tumor nursing, nursing teaching, and nursing management for ≥10 years; b) Title above supervisor nurse, bachelor's degree or above; and c) Mastered the nursing knowledge and skills of this major, and have rich experience in teaching management, and also has mastered the knowledge and skills of nursing, with a rich experience in teaching management; and d) actively participating in this research.

#### Implementation Process of Expert Inquiry

A questionnaire was distributed by a specially assigned person for the job and the experts were required to reply within 1 week. The experts scored and revised the importance of each index according to their own cognition and understanding of it. A total of 17 valid questionnaires were collected during the first letter of inquiry. Statistical analysis was performed on the results. In combination with the opinions of the experts, the items with the significant average score >3 points and the coefficient of variation <0.35 were retained. The questionnaire used in the first round was deleted, supplemented, and modified to form the second round of questionnaire, and the second round of expert consultation was conducted 2 weeks later. A total of 17 valid questionnaires were collected during the second consultation. After the analysis of the second round of questionnaires, it was found that the opinions of the experts were consistent, and hence the expert inquiry was ended.

### Statistical Methods

The SPSS 17.0 statistical software was used to analyze the data. The enthusiasm of experts was expressed by the effective recovery rate of the questionnaire, and the degree of expert authority was expressed by the authority coefficient. The degree of dispersion of expert opinions has been expressed by the coefficient of variation (CV) and coordination coefficient (*W*), where CV = standard deviation/mean value. The smaller the coefficient of variation, the more unanimous the opinion of experts. The value of *W* was statistically tested by the non-parametric test of multiple related samples. The significant value indicated good coordination after the test. *W* was in the range of 0–1, and the larger the *W* was, the better the coordination would be. The importance scores of each item were expressed as mean and SD ($\bar{x}\pm s$), and the weight of each item was determined by the analytic hierarchy process.

## Results

### General Information for 17 Experts

According to the requirements of the Delphi method for expert selection, combined with the specific characteristics and feasibility of this study, 17 nursing experts from the Level A tertiary hospitals were selected as the inquiry objects. They were from different professional departments, such as oncology, radiotherapy, thoracic surgery, gynecology and oncology, head and neck surgery, breast surgery, hepatobiliary surgery, pancreatic biology, colorectal surgery, orthopedics, neurosurgery, and nursing. The basic situation of the experts is shown in Table 1.

**Table 1:** General information for 17 experts.

**General information**		**Number**	**Constituent** **ratio (%)**
Age (years)	30–40	12	70.59
	41–50	4	23.53
	>50	1	5.88
Professional title	Nurse-in-charge	4	23.53
	Deputy director of the nurse	9	52.94
	Chief nurse	4	23.53
Academic degree	Undergraduate course	12	70.59
	Master	5	29.41
Working years (years)	10–15	4	23.53
	16–20	7	41.18
	>20	6	35.29

### Positive Coefficient of Experts

The expert positive coefficient refers to the attention and cooperation of experts in research. Experts' enthusiasm for this research is not high, which will affect the objectivity and reliability of the consultation. The expert's positive coefficient is expressed by the effective recovery rate of questionnaires. In the first and second rounds of this study, a total of 17 questionnaires were distributed and 17 valid questionnaires were returned. The questionnaire recovery rate was 100%, which showed that the experts were highly motivated.

### Authority of Experts (Cr)

**Figure 1:**
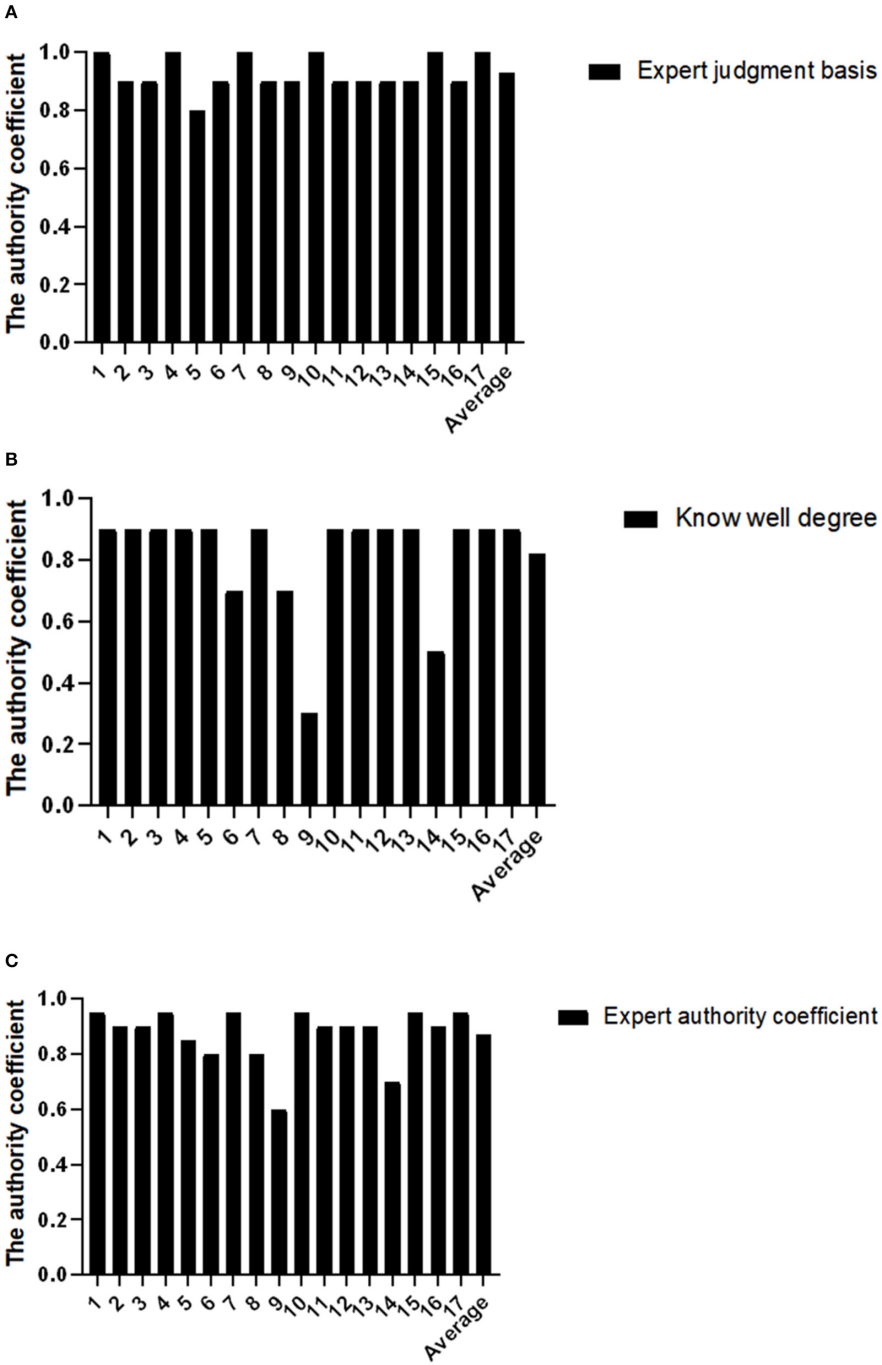
Degree of authority of the inquiry experts. **(A)** Expert judgment basis; **(B)** Know well degree; **(C)** Expert authority coefficient.

The authority coefficient of an expert is determined by the judgment basis coefficient (*Ca*) and familiarity coefficient (*Cs*) of the expert, and its calculation formula is *Cr* = (*Ca*+*Cs*)/2. The judgment basis coefficient, familiarity coefficient, and authority coefficient of the expert in this study are 0.93, 0.82, and 0.87, respectively, as shown in Figure 1. Generally, it is considered that *Cr* is greater than 0.7, the consulting result is reliable, and the prediction accuracy is improved with the improvement of an expert authority. Therefore, the degree of expert authority in this study was high, and the results were credible.

### Coordination of Expert Advice

The degree of dispersion of the expert inquiry was expressed by the coordination coefficient (*W*). The coefficient of variation of the whole index ranged from 0–0.25, all of which were less than 0.35. The range of the matching coefficient is 0–1. A larger *W* indicates a better coordination degree. The coordination coefficients of all levels are shown in Table 2. It can be seen from the table that the *W* values of the first, second, and third level indicators are 1.000, 1.000, and 0.978, respectively. The *P*-values calculated by the Chi-square test are all less than 0.05. Statistics show that experts have a high degree of recognition of all indicators, and the result is satisfactory.

**Table 2:** Test results of coordination degree of two rounds of expert inquiry.

**Number of consultations**	**Indicator** **hierarchy**	**Coordination factor (w)**	**Chi-square value**	***P value***
Round 1	Level 1 indicators	1.000	17.000	<0.001
	Level 2 indicators	1.000	68.000	<0.001
	Level 3 indicators	0.966	1,477.957	<0.001
Round 2	Level 1 indicators	0.235	4.000	0.046
	Level 2 indicators	1.000	68.000	<0.001
	Level 3 indicators	0.978	1,496.007	<0.001

### Results of Expert Communications

The degree of expert opinion set is expressed with the mean and SD of the index importance assignment. The larger the mean value of significant distribution, the smaller the SD, indicating the high importance of the index. The results of expert inquiry showed that the mean range of important assignments for the whole index was 3.44–5.00, and the SD range was 0–0.95. The small difference indicated that the expert opinion was centralized. After two rounds of expert consultation, it was finally determined that the training content system for new nurses in tumor specialized hospitals included two level I indicators, five level II indicators, and 45 level III indicators. Tables 3, 4 show the specific contents.

**Table 3:** Results of level I and level II indexes of new nurse training content system.

**Indicator code**	**Indicator name**	**Importance assignment**	**Variable coefficient**	**Weight**
I-1	Knowledge skills	4.94 ± 0.25	0.051	0.510
I-2	Comprehensive ability accomplishment	4.75 ± 0.45	0.095	0.490
II-1	Nursing theory knowledge	4.81 ± 0.40	0.083	0.491
II-2	Common nursing operation technology	5.00 ± 0.00	0.000	0.510
II-3	Ability of practice	4.69 ± 0.48	0.102	0.326
II-4	Emergency capability	4.88 ± 0.34	0.070	0.339
II-5	Professional quality	4.81 ± 0.40	0.083	0.335

**Table 4:** New nurse training content system level III index results.

**Indicator code**	**Indicator name**	**Importance assignment**	**Variable coefficient**	**Weight**
I-1	Knowledge skills			
II-1	Nursing theory knowledge			
III-1	First-aid knowledge	4.88 ± 0.34	0.070	0.089
III-2	Nursing safety management	4.81 ± 0.40	0.083	0.088
III-3	Occupational protection knowledge	4.81 ± 0.40	0.083	0.088
III-4	Hospital infection prevention control knowledge	4.75 ± 0.45	0.095	0.087
III-5	Nursing core system	4.81 ± 0.54	0.112	0.085
III-6	Nursing norm standard	4.75 ± 0.68	0.143	0.080
III-7	Tumor nursing specialist knowledge	4.44 ± 0.89	0.200	0.076
III-8	Basic nursing knowledge	4.44 ± 0.73	0.164	0.074
III-9	Nursing related laws regulations	4.19 ± 0.83	0.198	0.071
III-10	Hospital regulations	4.31 ± 0.95	0.220	0.070
III-11	Oncology specialist knowledge	4.06 ± 0.85	0.209	0.065
III-12	Psychological nursing	4.00 ± 0.82	0.205	0.064
III-13	Standardized treatment of pain	3.94 ± 0.77	0.195	0.063
II-2	Common nursing operation technology			
III-14	Emergency nursing technique	5.00 ± 0.00	0.000	0.271
III-15	Basic nursing technology	4.63 ± 1.03	0.222	0.247
III-16	Tumor specialist nursing technology	4.63 ± 0.62	0.134	0.241
III-17	Instrument equipment use	4.63 ± 0.62	0.134	0.241
I-2	Comprehensive ability accomplishment			
II-3	Ability of practice			
III-18	Condition observation	4.63 ± 0.72	0.156	0.085
III-19	Symptom care	4.56 ± 0.73	0.160	0.083
III-20	Nursing document writing	4.56 ± 0.63	0.138	0.083
III-21	Information system operation	4.56 ± 0.63	0.138	0.083
III-22	Teamwork	4.50 ± 0.82	0.182	0.080
III-23	Communication	4.44 ± 0.73	0.164	0.081
III-24	Health education	4.38 ± 0.81	0.185	0.080
III-25	Nursing rounds	4.25 ± 0.78	0.184	0.078
III-26	Autonomous learning	4.25 ± 0.68	0.160	0.078
III-27	Discussion on nursing problems	4.00 ± 0.63	0.158	0.073
III-28	Workplan co-ordination capacity	4.00 ± 0.82	0.205	0.071
III-29	Critical thinking ability	3.81 ± 0.83	0.218	0.067
III-30	Ward management	3.44 ± 0.89	0.259	0.058
II-4	Emergency capability			
III-31	Emergency ability to use drugs treat adverse reactions	4.94 ± 0.25	0.051	0.148
III-32	Patient safety event emergency response capability	4.88 ± 0.34	0.070	0.146
III-33	Emergency ability to cope with sudden changes in illness	4.88 ± 0.50	0.102	0.141
III-34	Emergency treatment ability of chemotherapeutic drug extravasation	4.81 ± 0.40	0.083	0.150
III-35	Emergency treatment ability of chemotherapeutic drug overflow	4.75 ± 0.45	0.095	0.143
III-36	Fire safety emergency capability	4.63 ± 0.50	0.108	0.139
III-37	Ability to handle emergencies such as water power outages	4.63 ± 0.62	0.134	0.133
II-5	Professional quality			
III-38	Professional ethics	4.88 ± 0.34	0.070	0.141
III-39	Solitary spirit	4.88 ± 0.34	0.070	0.141
III-40	Medical ethics	4.75 ± 0.58	0.122	0.132
III-41	Emotional control	4.69 ± 0.60	0.128	0.130
III-42	Humanistic care	4.50 ± 0.73	0.162	0.119
III-43	Nurse etiquette	4.31 ± 0.70	0.162	0.114
III-44	Courtyard view culture	4.25 ± 0.86	0.202	0.114
III-45	Professional identity planning	4.25 ± 0.93	0.219	0.109

## Discussion

### The Scientificity and Reliability of This Study

In the Delphi expert inquiry method, the selection of experts is related to the rationality and reliability of the research results (7). In the process of selecting experts for this study, nursing experts from departments such as oncology, internal medicine, radiotherapy, thoracic surgery, gynecology and oncology, head and neck surgery, breast surgery, liver and gallbladder surgery, pancreas and stomach surgery, colorectal surgery, orthopedics, neurosurgery, and nursing were selected and could continue to participate in the two rounds of consultation in this study. These experts had high academic levels and rich clinical experience in relevant fields, ensuring the scientificity and authority of research results.

The results obtained from this study showed that the recoveries of the two rounds of questionnaires were 100%, indicating that the enthusiasm of experts was high. The expert's judgment basis coefficient, familiarity coefficient, and authority coefficient are 0.93, 0.82, and 0.87, respectively, which are all greater than 0.7, indicating that the expert's authority is high. The *W* values of the indicators in the second round were 1.000, 1.000, and 0.978, respectively. The *P*-values calculated by the Chi-square test were all less than 0.05, showing statistical significance, indicating that experts had a high degree of approval for all indicators. These research results indicated the reliability and scientificity of the evaluation index system.

### The Content of New Nurse Training System in Tumor Specialized Hospital Based on Competency

After two rounds of the Delphi expert consultation, the content of a competency-based training system for new nurses in cancer hospitals was finally established in this study. There were two level I indicators: knowledge and skills and comprehensive ability and accomplishment, five level II indicators: nursing theoretical knowledge, common operation technology, practical ability, emergency ability, and professional quality. The results showed that in the training system, the common nursing operation technology (0.510) ranked first in the weight value of the level II, and its level III indicator included basic nursing technology, tumor specialized nursing technology, emergency nursing technology, and the use of instruments and equipment. The weight of common nursing operation techniques was higher than that of nursing theoretical knowledge, which might be related to the fact that the new nurses had just finished their school study career, and their theoretical knowledge was relatively solid, but their operation skills were relatively weak. The highest weight in the three indicators was emergency care technology (0.271). As the number of critically ill patients increases, the management of critically ill patients becomes more challenging, says Kaldan (8). High-quality critical care practice is essential for critical patient management. Research results showed that the ability to provide emergency treatment and cooperate with emergency treatment for critically ill patients was urgently needed in clinical practice, but training in emergency techniques was not widely available during the study period (9). Therefore, the weight of emergency nursing technology, as an important influencing factor of critical patient management and urgently needed work ability in clinical practice, is in the first place of the level III indicators. The nursing theoretical knowledge (0.491), which included 13 parts such as rules and regulations, core system, basic knowledge, and specialized knowledge, ranked second in the weight of secondary indicators. Tariman's research shows that knowledge is one of the main factors affecting the ability of nurses specializing in the treatment of tumors to work (10). Therefore, the theoretical knowledge has a higher weight in the new nurse training system of tumor hospitals. The first-aid knowledge (0.089) had the highest weight in the level III indicators, which was consistent with the result that the weight of emergency care technology was in the first place. The knowledge related to emergency treatment is of high importance in tumor hospitals, which may be related to the poor general condition of tumor patients, urgent condition change, and the rapid development of disease courses. The weights of nursing safety management, occupational protection knowledge, hospital infection prevention and control knowledge, nursing core system, and nursing specification standard in the three-level indicators were located at a higher level (0.080–0.088). Considering that these contents were closely related to the daily nursing work, it was the bottom line of nursing work, and any mistake would cause adverse consequences, so the importance was higher. The laws and regulations related to nursing (0.071) and hospital rules and regulations (0.070) in the level III indicators included the duties and rights of nurses. The duties of nurses were clearly reflected in the core system of nursing, nursing norms and standards, and other related contents. However, the rights of nurses had a low degree of a close relationship with clinical adverse events. Therefore, the weights of nursing laws and regulations and hospital rules and regulations were lower than those of nursing safety management. The weights of psychological care and standardized pain treatment (0.064 and 0.0629) were relatively low. Compared with these two contents, emergency knowledge and other issues were considered, which was related to the low urgency of clinical needs. However, the hospice care and symptom care for cancer patients involve psychological and pain related contents, so the relevant training contents were retained in two rounds of an expert consultation. The third important factor in the weight of secondary indicators was emergency response ability, which included adverse drug reactions, chemotherapy extravasation, safety and fire protection, etc. Patient safety is the primary content of medical work, and the emergency ability of nurses is closely related to the clinical outcome and safety problems of patients. However, the emergency ability of new nurses is generally weak (11). Strengthening the emergency ability of new nurses can effectively improve the competency (12). The highest weight of the level III indicators was emergency response ability to medication and adverse reaction after treatment (0.148), which included medication error, anaphylactic shock, infusion and blood transfusion reaction, etc. Medication and treatment are frequently performed by nurses every day, and adverse reactions have immediate and severe consequences. For example, patients undergoing chemotherapy are prone to drug allergic reactions due to drug accumulation in the body at the later stage of treatment. With the increase in treatment cycles, the allergic reactions gradually aggravate. Therefore, nurses need to have a certain emergency ability to ensure smooth treatment of patients and treatment safety (13, 14). The professional accomplishment (0.335) ranked fourth in the weight of secondary indicators. Nursing is a subject that not only pays attention to the practical operation but is also a specialty with high requirements for professional accomplishment. The professional ethics and self-caution in the level III indicators were ranked first in the weight (0.141). Regular nurses who attach importance to the cultivation of professional ethics have a high degree of awareness of their own professional behavior and the teachers have a high degree of satisfaction with their work performance (15). According to Monroe's research, nurses who had worked for less than 10 years had low scores of professional values, and the cultivation of professional ethics could improve the professional values of the nurses, improve the quality of nursing work, and reduce the turnover rate (16). The cultivation of professional ethics and the spirit of self-caution is conducive to improving the quality of nursing work and the professional values of new nurses. The weight of emotional control (0.130) was higher than that of humanistic care and nurse etiquette. Considering that the emotions of cancer patients were generally poor, nurses contacted patients most frequently before their death. When faced with the dying state of the patients they were nursing, the nurses would have psychological fluctuations such as regret, anxiety, and fear (17, 18). Emotional control can reduce the negative emotions of nurses and improve their enthusiasm in dealing with work. The occupational identity and planning weights of the level III indicators were the lowest (0.109), which was considered related to the ability that was not considered as a clinically urgent need. However, the study has pointed out that the professional identity has a positive correlation with the competency and job performance of new nurses (19). Factors that affected the professional identity of new nurses included educational background, internship time, labor and personnel relations, etc. It is recommended that targeted training programs be adopted for new nurses with different academic qualifications (20). Through systematic and targeted training, we can improve the professional identity of new nurses and ultimately improve the post-competency. Practical ability (0.326) ranked fifth in the weight of secondary indicators, including condition observation, file writing, system operation, critical thinking, and ward management. The content with the lowest weight of the level was ward management (0.058), but ward management is not only the responsibility of the management position, nurses need to provide patients, families, and other medical staff with nursing related knowledge, also need to effectively take charge of the daily management affairs of the ward and to ensure the smooth progress (21). A good ward manager should have solid knowledge theory, operation technology, good overall planning ability, and communication ability, etc., can do a good job in the ward management, so for new nurse training ward management cannot as the focus, suggestions in the follow-up stratified training to follow up.

## Conclusion

In the new nurse training content system based on post-competency in cancer hospital constructed in this study, the expert opinions of evaluation indicators at all levels tend to be consistent, with high credibility and a certain degree of science and authority, which can provide a reference for the training of new nurses in the cancer hospital. This study did not test the effectiveness and effect of the training content system. Therefore, the effect of the study of this training content system will be the main content of the next study. It was suggested that hospice care and spiritual care should be included in future research so that new nurses with different academic qualifications could receive targeted training according to the difficulty of the training content.

## Data Availability Statement

The original contributions presented in the study are included in the article/supplementary materials, further inquiries can be directed to the corresponding author/s.

## Ethics Statement

Ethical review and approval was not required for the study on human participants in accordance with the local legislation and institutional requirements. Written informed consent for participation was not required for this study in accordance with the national legislation and the institutional requirements.

## Author Contributions

ML and JGe are mainly responsible for data statistics and paper writing. JGa and ZM are mainly responsible for data search and training system construction. XW and SW are mainly responsible for designing schemes and overall research. YL is mainly responsible for the guidance of the entire research. All authors contributed to the article and approved the submitted version.

## Funding

This study was supported by Special Fund for Hospital Management Research Project LC2018D04.

## Conflict of Interest

The authors declare that the research was conducted in the absence of any commercial or financial relationships that could be construed as a potential conflict of interest.

## Publisher's Note

All claims expressed in this article are solely those of the authors and do not necessarily represent those of their affiliated organizations, or those of the publisher, the editors and the reviewers. Any product that may be evaluated in this article, or claim that may be made by its manufacturer, is not guaranteed or endorsed by the publisher.
